# Effect of a fixed dose of fresh frozen plasma on systemic inflammation and endothelial damage in nonbleeding critically ill patients

**DOI:** 10.1186/cc13306

**Published:** 2014-03-17

**Authors:** M Straat, M Muller, J Meijers, M Schultz, N Juffermans

**Affiliations:** 1 Academic Medical Center, Amsterdam, the Netherlands

## Introduction

Fresh frozen plasma (FFP) is associated with onset of acute lung injury [1], the mechanism of which is largely unknown. On the other hand, FFP may be beneficial, as a higher ratio of FFP to red blood cells decreases mortality in bleeding trauma patients [2] and is associated with an endothelial stabilizing effect *in vitro *[3]. We investigated the effect of transfusion with FFP on host response and markers of endothelial damage in nonbleeding critically ill patients.

## Methods

This was a substudy of a multicenter trial in which nonbleeding critically ill patients with an increased International Normalized Ratio (1.5 to 3.0) were randomized to omitting or administering a prophylactic transfusion of FFP (12 ml/kg) prior to an invasive procedure. In 38 patients randomized to receive FFP transfusion, we measured levels of factor VIII, von Willebrand factor, and markers of proinflammatory response before and after transfusion. Data are presented as medians.

## Results

FFP transfusion resulted in a significant decrease of TNFa (from 12.3 to 3.1 pg/ml, *P *= 0.01), von Willebrand factor (from 475 to 424%, *P *< 0.01) and factor VIII (from 246 to 244%, *P *< 0.01). FFP did not alter levels of IL-1β, IL1-RA, IL-8, IL-10, MCP1, MIP1A or sCD40L. Patients had some degree of lung injury at baseline as reflected by a lung injury score of 2 (0.8 to 2.5), which did not change following transfusion. None of the patients developed TRALI.

## Conclusion

FFP transfusion is not associated with a proinflammatory response in the critically ill. Rather, FFP seemed to have an endothelial stabilizing effect.
